# Bruton tyrosine kinase (BTK) may be a potential therapeutic target for interstitial cystitis/bladder pain syndrome

**DOI:** 10.18632/aging.204271

**Published:** 2022-09-05

**Authors:** Guang Wang, Tong-Xin Yang, Jiong-Ming Li, Zi-Ye Huang, Wen-Bo Yang, Pei Li, Da-Lin He

**Affiliations:** 1 Department of Urology, The First Affiliated Hospital of Xi’an Jiaotong University, Xi’an 710061, Shanxi, China; 2 Department of Urology, The Second Affiliated Hospital of Kunming Medical University, Kunming 650101, Yunnan, China

**Keywords:** interstitial cystitis, bioinformatics, biomarker, mast cells, BTK

## Abstract

Aims: To determine the potential diagnostic and therapeutic targets of Interstitial Cystitis/Bladder Pain Syndrome (IC/BPS).

Methods: We selected the GSE11783, GSE57560 and GSE621 datasets from the GEO database and merged them. R software was used to screen differentially expressed genes (DEGs) between IC/BPS and normal bladder tissues. The "String" online tool is used to analyze DEGs interaction and functional protein enrichment. CIBERSORT online tool was used to analyze the infiltration of immune cells. In addition, we verified the function of BTK in IC/BPS at the clinical samples and cells level.

Results: Bioinformatics analysis revealed that 5 genes were significantly overexpressed in IC/BPS, and the protein-protein interaction diagram showed that BTK was a critical link between these five proteins. At the same time, functional enrichment showed that they were significantly related to innate immunity. Immunoinfiltration showed that mast cell resting in IC/BPS was significantly higher. IHC staining of clinical samples showed that the mast cell markers Tryptase and BTK were highly expressed in IC/BPS tissues. At the cell level, knockdown of BTK inhibited proliferation, migration, invasion, and degranulation of mast cells.

Conclusions: This study provides a new perspective for understanding the molecular mechanisms involved in IC/BPS and suggests that BTK may be a target for treating IC/BPS.

## INTRODUCTION

Interstitial Cystitis (IC) is a chronic non-bacterial inflammatory bladder disease of unknown cause. It is mainly manifested as bladder area or lower abdomen, suprapubic pain and/or frequency, urgency and other clinical symptoms [1]. The incidence of IC is related to race, region, gender and other factors. According to statistics, the incidence of female patients in the United States is 2.7%-6.53%, that of female patients in Japan is about 3-4/100,000, and that of female patients in Europe is about 18/100,000, among which the incidence rate of female patients is about 5 times that of male patients, and the data shows an increasing trend year by year [2].

The diagnosis of IC has long been a difficult problem for urologists, because it does not have a "golden standard" for diagnosis [3]. At present, the diagnosis of this disease tends to clinical symptoms. In order to overcome the differences between clinical practice and scientific research, it is necessary to establish a unified clinical diagnosis standard. In 1987, the National Institute of Diabetes and Digestive and Kidney Diseases (NIDDK) of the United States formulated the experimental diagnostic standard of IC. However, this standard is too strict. According to this standard, two thirds of patients diagnosed with IC in clinic fail to meet the diagnostic standard, which is mainly applicable to scientific research [4]. Therefore, in 2005, the European Society for the Study of Interstitial Cystitis (ESSIC) introduced a new definition: Bladder Pain Syndrome (BPS), which was described as "suprapubic pain associated with bladder filling, accompanied by other symptoms (such as frequent urination during the day and night) and lack of urinary tract infection or other pathological evidence" [5]. In addition, the Society for Urodynamics and Female Urology [6] and the American Urological Association [2] have also given different definitions for IC/BPS. All these indicate that IC/BPS is difficult to be diagnosed clinically. The pathogenesis of IC/BPS is not clear, and it is affected by many etiologies and caused by one or more pathogenic pathways. At present, the main etiological theories of IC/BPS include glycosaminoglycan theory, leaky epithelium theory, autoimmunity theory, mast cell and neurogenic inflammation theory or a combination of the above theories [7, 8]. However, many theories about its pathogenesis lack exact clinical evidence, and evidence of evidence-based medicine has not yet been found.

Mast cells are involved in immune regulation by secreting a variety of cytokines. More and more studies have found that the increase of mast cells in IC/BPS is closely related to bladder pain and inflammation [9–11]. Martin and colleagues [9] found that in IL-37-induced IC/BPS model mice, mast cell deficient mice exhibited lower inflammation levels and decreased pain responses. However, the regulatory mechanism of mast cell abnormalities in IC/BPS is not clear.

Because the pathogenesis of IC/BPS is not fully understood, it will take a long time to determine the diagnosis, and there is no direct treatment method at present. Therefore, it is urgent to find the diagnosis and treatment targets related to IC/BPS. The purpose of this study aims to provide potential targets for clinical diagnosis and treatment of IC/BPS through bioinformatics methods and further pathological and cytological validation.

## RESULTS

### Identification of DEGs in GEO datasets

**Figure 1:**
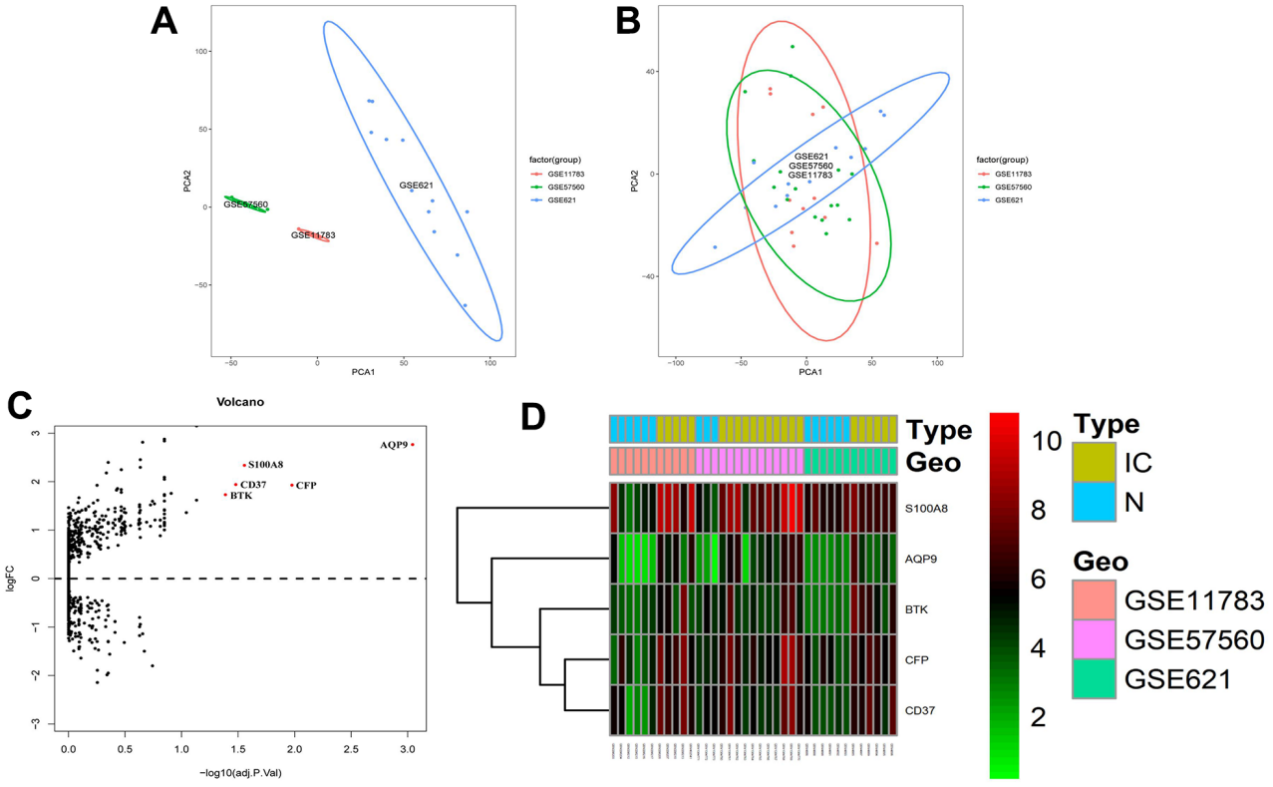
**Identification of DEGs in GEO dataset of IC/BPS patients.** (**A**, **B**) The ggplot2 package performed principal component analysis (PCA) on the (**A**) unnormalized and (**B**) normalized GSE11783, GSE57560 and GSE621 datasets. (**C**) Volcano maps of the merged datasets. (**D**) Heatmaps of cluster analysis by DEGs expression in GSE11783, GSE57560 and GSE621 datasets.

As shown in Figure 1A, 1B, the PCA analysis results of the GSE11783, GSE57560 and GSE621 datasets before and after normalization, respectively. Supplementary Tables 1, 2 showed detailed information about the three GEO datasets. Based on the screening criteria of adjusted *P*-Val < 0.05 and absolute logFC > 1, we obtained 5 up-regulated genes (S100A8, AQP9, BTK, CFP and CD37) and 0 down-regulated genes (Figure 1C, 1D). Supplementary Table 3 showed the filtered DEGs details.

### PPI and functional enrichment analysis

**Figure 2:**
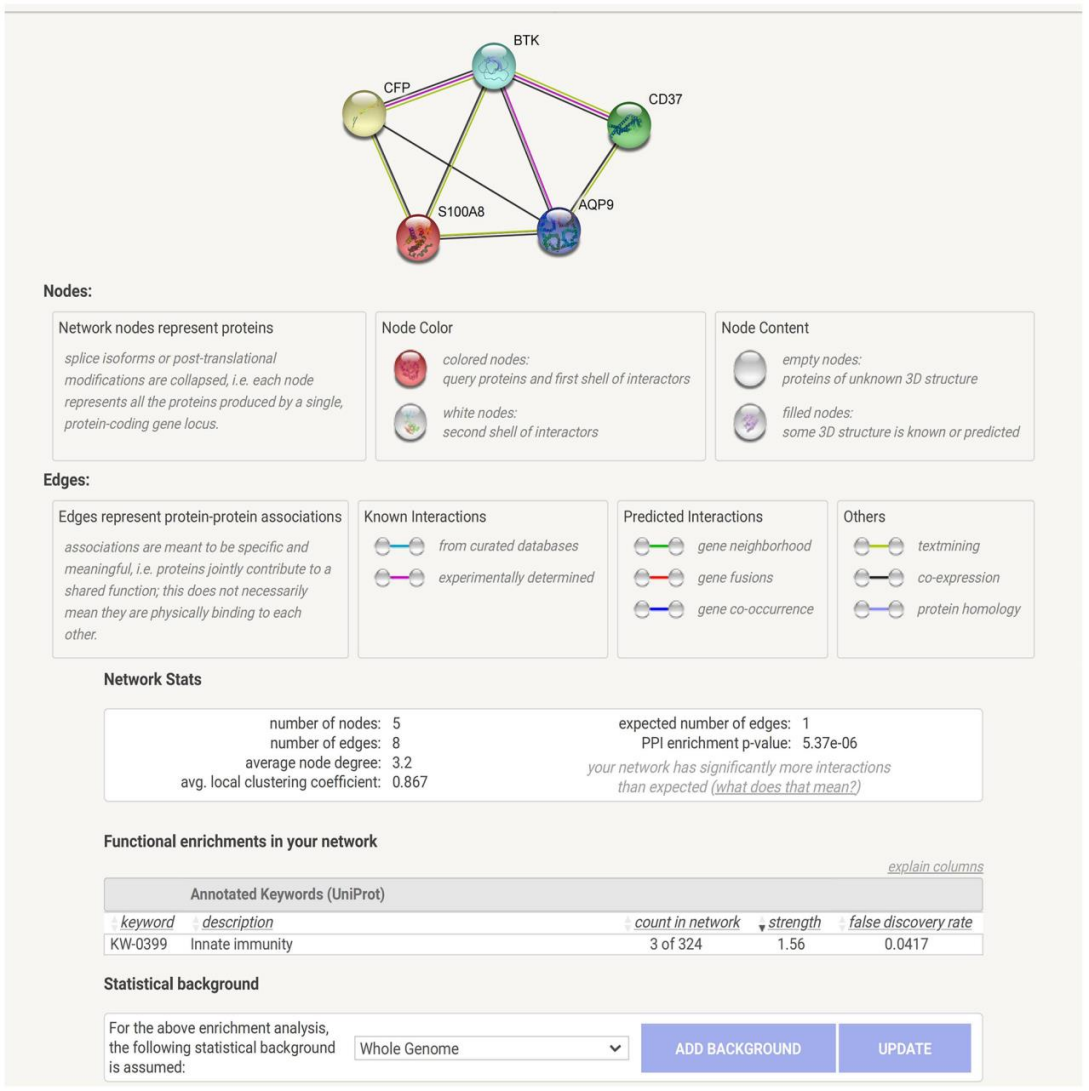
PPI and functional enrichment analysis.

PPI showed that these five proteins (S100A8, AQP9, BTK, CFP and CD37) were correlated with each other, and BTK was the central protein of this association. Meanwhile, functional enrichment showed that they were significantly related to innate immunity. (Figure 2, permission and/or credit for reproduced image: https://cn.string-db.org/cgi/access?footer_active_subpage=licensing).

### Immune-infiltration analysis

**Figure 3:**
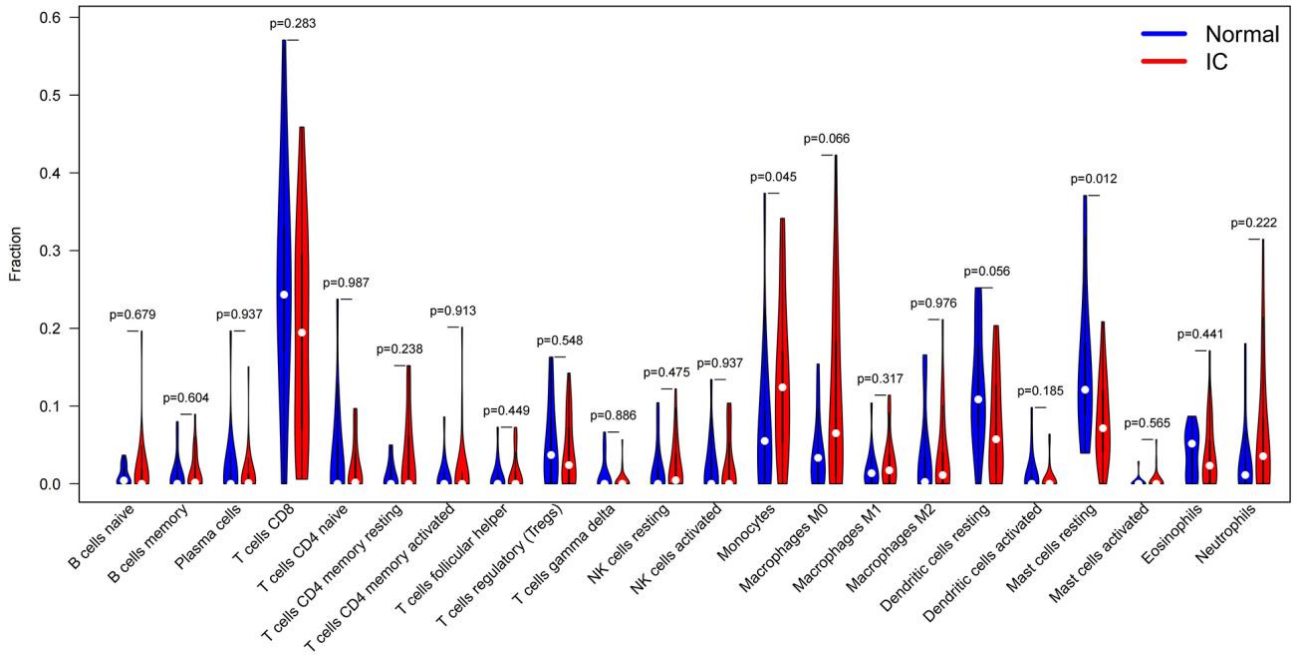
**Immune-infiltration analysis of GEO datasets of IC/BPS patients by the CIBERSORTx online tool.** Violin Plot and were used to compare the immune cell score difference between IC/BPS and normal bladder tissues (Blue indicates normal bladder tissues; red indicates IC/BPS bladder tissues. Wilcoxon signed rank test was used to compare and calculate the statistical p-value).

The results showed that the majority of the cell proportions in the IC/BPS group were not altered significantly compared to the control group in 22 immune cell types, but only mast cells resting and monocytes have significant differences (Figure 3, *P* < 0.05). And the difference of mast cells resting is more significant (*P* = 0.012), while the *P* value of monocytes is 0.045. Therefore, we will further study the mast cells of bladder wall tissue in IC/BPS patients.

### Differential expression of BTK in IC/BPS clinical samples

**Figure 4:**
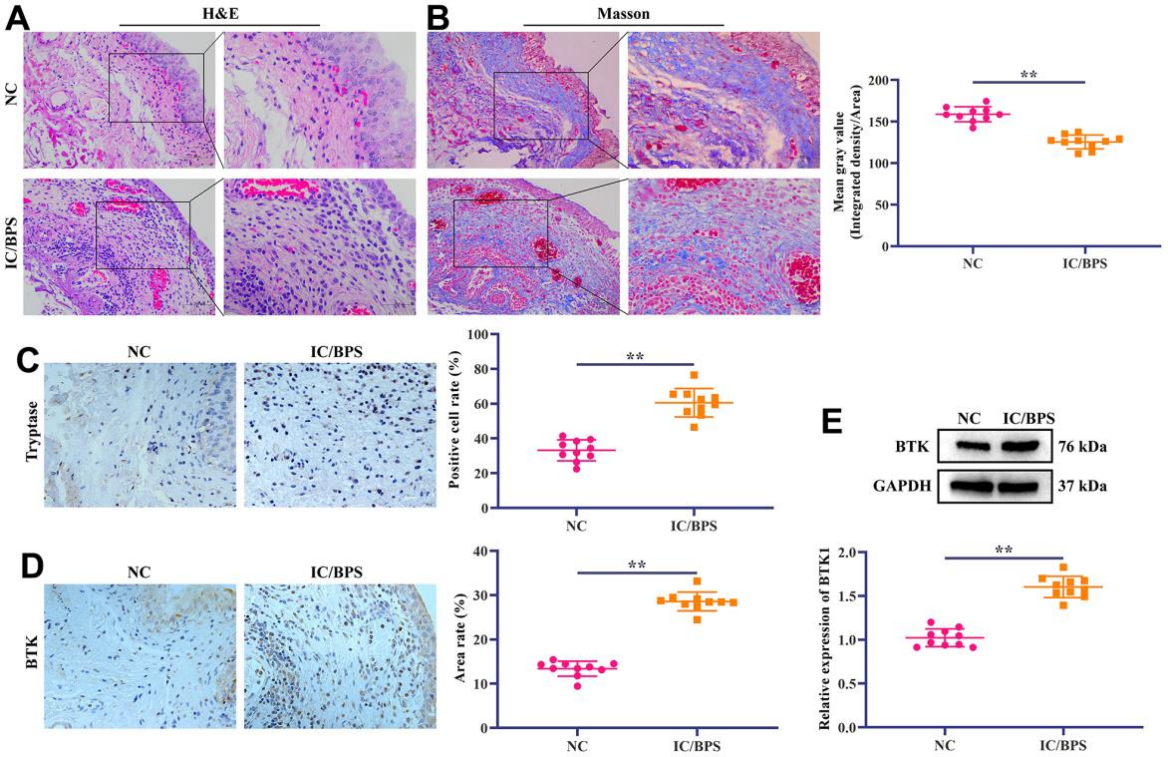
**Differential expression of BTK in IC/BPS clinical samples.** (**A**) HE staining shows the pathological state of IC/BPS samples. Scale bar: 100 and 50 μm. (**B**) Masson staining is used to distinguish collagen fibers from muscle fibers. Collagen fibers are blue and smooth muscle fibers are red. The staining results show that compared with NC group, collagen fibers in IC/BPS group are significantly reduced. Scale bar: 100 and 50 μm. (**C**, **D**) IHC staining showed the difference of (**C**) Tryptase and (**D**) BTK expression in normal and IC/BPS tissues. Scale bar: 20 μm. (**E**) Western blotting exhibited the expression level of BTK in normal and IC/BPS tissues. In all cases, Values are mean ± SD (n=10 for each group; *P<0.05, **P<0.01).

The results of HE staining showed that the mucous layer of normal bladder tissue was thick, the lamina propria was free of edema, the muscle fibers were abundant, the detrusor was clear, and no inflammatory cell infiltrated in the muscle bundles. In the IC/BPS bladder tissue, mucosa epithelium became thinner, the mucosa and lamina propria swelled, and chronic inflammatory cell infiltration was observed in the muscular bundle. The muscular bundle was hair-like, and muscle fibre atrophy (Figure 4A). Masson staining showed that smooth muscle fibers in the normal bladder tissues were well arranged, the bladder wall muscle fibers were abundant, the cells were full, and a few fibers were deposited between the detrusor muscle bundles. In the IC/BPS bladder tissue, smooth muscle fibers were disordered with detrusor muscular atrophy, and there were many fiber bundles and inflammatory cell infiltrated between muscle bundles (Figure 4B). In addition, the analysis of Image J software found that the collagen fiber content in IC/BPS bladder tissue was significantly lower than that in normal bladder tissues (Figure 4B). We further detected the level of Tryptase, a marker of mast cells, in the samples collected by IHC. The results showed that compared with NC group, the positive rate of tryptase in IC/BPS tissue was significantly higher (Figure 4C). The above staining results are consistent with the pathological results of bladder tissue in patients with IC/BPS. Because BTK plays an important role in regulating mast cells in other diseases, the mechanism of BTK in IC/BPS and its influence on mast cells still need to be studied. Therefore, we chose BTK in DEGs for verification. IHC staining showed that the positive rate of BTK protein in IC/BPS tissue was significantly higher than that in NC group (Figure 4D). The Western blotting also got consistent results (Figure 4E). It indicates that the high expression of BTK may be involved in the occurrence of IC/BPS.

### *In vitro* verification of the effect of BTK on biological behavior of HMC-1 cells

**Figure 5:**
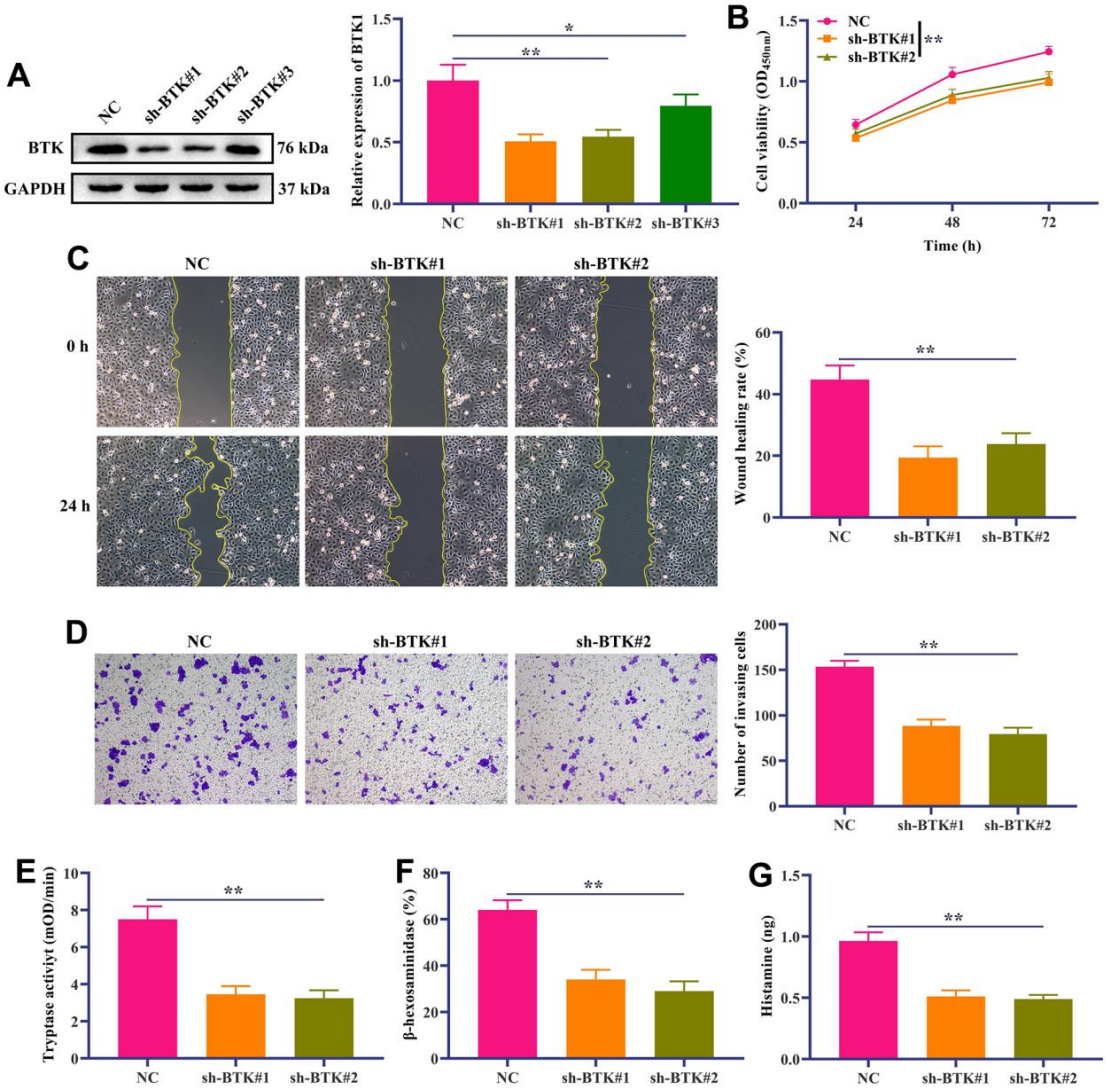
***In vitro* verification of the effect of BTK on the proliferation and metastasis of HMC-1 cells.** (**A**) Western blotting displayed the knockdown efficiency of sh-BTK. (**B**) HMC-1 cell proliferation was detected by CCK-8 kit. (**C**) Wound-Healing assay showed the migration ability of HMC-1 cells. Scale bar: 100 μm. (**D**) Transwell assay was used to detect HCC-1 cell invasion. Scale bar: 100 μm. (**E**–**G**) Quantifications of mast cell mediators, including (**E**) Tryptase, (**F**) β-hexosaminidase and (**G**) Histamine. In all cases, Values are mean ± SD (n=3 for each group; *P<0.05, **P<0.01).

Cumulative studies have shown that abnormal activation and chemotaxis of mast cells are one of the main pathological mechanisms of IC/BPS [1]. Therefore, we further explored the effect of BTK on mast cell proliferation, metastasis and degranulation of mast cells at cellular level. At first, we transfected sh-BTK into stimulated HMC-1cells to knock down the expression of BTK. Western blotting results showed that compared with the normal group, the three sh-BTKs could significantly down-regulate the expression level of BTK in HMC-1 cells, among which sh-BTK #1 and sh-BTK #2 had the best effects (Figure 5A). We chose sh-BTK #1 and sh-BTK #2 for follow-up experiments. The results of CCK-8 showed that the proliferation of HMC-1 cells in the sh-BTK #1 and sh-BTK #2 groups was significantly lower than that in the normal group (Figure 5B). It suggests that BTK can regulate the proliferation of HMC-1 cells. Furthermore, we also used Wound-Healing and Transwell assay to study the effect of BTK on migration and invasion of HMC-1 cells. The results showed that, compared with the normal group, the Wound healing rate of HMC-1 cells in the sh-BTK #1 and sh-BTK #2 groups was significantly lower (Figure 5C). As expected, the number of HMC-1 cell migration in the sh-BTK #1 and sh-BTK #2 groups was significantly lower than that in the normal group (Figure 5D). We further studied the effect of BTK on the degranulation of activated HMC-1 cells. The results showed that compared with the normal group, the levels of Tryptase, β-hexosaminidase and Histamine in the supernatant of HMC-1 cells in the sh-BTK #1 and sh-BTK #2 groups were significantly reduced (Figure 5E–5G). These results indicated that BTK can promote the proliferation, migration, invasion and degranulation of HMC-1 cells.

## DISCUSSION

IC/BPS is a clinical syndrome characterized by frequent urination, urgency, pain in suprapubic area after bladder filling, and relief after urination with unknown etiology and pathogenesis. There are many hypotheses about the etiology of IC/BPS, which mainly focus on the study of bladder mucosal epithelial function and injury mechanisms, such as the decrease of glycosaminoglycan in mucosa, the activation of mast cell, neurotransmitters, pain and local autoimmunity [12, 13]. In this study, 5 up-regulated DEGs (AQP9, CFP, S100A8, CD37 and BTK) were identified through bioinformatics methods. PPI showed that BTK was the central protein of the correlation, so we selected BTK for subsequent validation. We found that BTK is abnormally overexpressed in the bladder tissues of patients with IC/BPS and promoted the proliferation, invasion, migration, and degranulation of mast cell *in vitro*. It is suggested that BTK can be used as a target for diagnosing and treating IC/BPS.

In addition, we analyzed the immune infiltration of 22 kinds of immune cells in the merged dataset and found that mast cells resting in IC/BPS was significantly higher. Mast cells mainly exist in connective tissue and mucosa epithelium, usually scattered around the capillaries of the skin, respiratory tract and digestive tract, which are easy to contact with the outside world. Mast cells play an important role in regulating and monitoring the inflammatory reaction, innate immune response and IgE-related immediate allergic reactions of the body. The cytoplasmic granules of mast cells contain histamine, serotonin, various cytokines and trend factors, platelet active factors, etc. These mediators have biological functions, regulating intestinal smooth muscle, nervous function and allergic reactions of the body [14–16]. When the antigen binds to the antibody, the receptor molecules on the surface of mast cells aggregate, resulting in the release of inflammatory mediators stored in the cytoplasmic granules to the extracellular domain, which is called degranulation [17]. At present, mast cells are considered to play an important role in the development, persistence and pain related to IC/BPS. Malik and colleagues showed that the number of mast cells in the bladder of patients with IC/BPS increased significantly, and the related mediators secreted by mast cells in the urine also increased significantly, such as IL-6, NGF, prostaglandin D2, histamine and trypsin, etc. [18]. Because these mediators are vasoactive, painful and pro-inflammatory, mast cells are considered to play an important role in the pathophysiology of IC/BPS [19]. Neuropeptides (such as substance P and neurotensin), NGF, TNF-α, SCF and other factors can activate mast cells to release mediators. For example, in patients with IC/BPS, SCF produced by dysfunction or injury of urothelial cells can promote the activation of mast cells [20]. Christmas reported that the density of nerve fibers in patients with IC/BPS increased significantly, and most newly proliferated nerve fibers contained substance P [21]. Substance P is an effective stimulator for mast cell degranulation, which can stimulate mast cells to release histamine and feedback the release of SP. These compounds can increase the sensitivity of sensory neurons, thus forming a positive feedback loop, further activates mast cells and maintaining the sustained and rapid increase of inflammatory cytokines release [22]. Although the relationship between the number and nature of mast cells and the pathogenesis of IC/BPS is still unclear, their degranulation may be the main cause of bladder pain, frequent urination and urgency in patients with IC/BPS.

BTK is a cytoplasmic protein expressed in hematopoietic cells, and a member of the Tec family of non-receptor protein tyrosine kinases. It can specifically bind to its receptors to activate downstream substrates and activate a variety of downstream signaling pathways, which play an important role in regulating cell proliferation, differentiation and apoptosis [23]. BTK is associated with the development of X-linked agammaglobulinemia [24], lymphoma [25], myeloma [26] and arthritis [27]. Recent studies have shown that BTK can significantly regulate various biological behaviors of mast cells. Carolin and colleagues showed that BTK is a central positive regulator of antigen-triggered mast cell activation, which can control Ca^2+^ mobilization, degranulation and cytokine production [28]. Melanie and colleagues found that irreversible BTK inhibitors can widely prevent lgE-mediated degranulation of human mast cells and cytokine production, and prevent allergen-induced isolated human bronchoconstriction [29]. In canine mast cell tumors, Ibrutinib, a BTK inhibitor, can inhibit IgE-dependent activation and histamine release in mast cells [30].

In contrast, Huang and colleagues showed that Btk(-/-) mast cells showed highly active preformation and LPS-induced TNF-α production [31]. Hye and colleagues also showed that BTK is essential for the ability of antigens to amplify the chemotactic response of mast cells by regulating the signal pathway that controls the rearrangement of F-actin [32]. However, the regulation of BTK on the biological behavior of mast cells in IC/BPS remains to be studied. In this study, we found through bioinformatics analysis that BTK is an up-regulated DEG in the tissues of IC/BPS patients. In addition, our preliminary discussion at the clinical and cellular level also found that BTK is highly expressed in IC/BPS tissues, and BTK can promote the proliferation, invasion, migration and degranulation of mast cells. In this study, we co-stimulated mast cell HCM-1 with anti-TNP IgE, OVA-TNP, and Ca^2+^ carrier as an *in vitro* model of IC/BPS. However, no consensus has been reached on the induction method of IC/BPS *in vitro* model, and the simulation of IC/BPS by activating mast cells is still controversial, which requires our in-depth exploration in future studies to further clarify the *in vitro* induction method of IC/BPS.

## CONCLUSIONS

This study provides a new perspective for understanding the molecular mechanisms involved of IC/BPS, and suggests that BTK may be a target for diagnosis and treatment of IC/BPS.

## MATERIALS AND METHODS

### IC/BPS GEO datasets information

mRNA transcriptomic profiles downloaded from GEO database (https://www.ncbi.nlm.nih.gov/geo/). The keyword ‘Interstitial cystitis/Bladder Pain Syndrome’ in the GEO database was searched to download the gene expression profile data. We included 24 IC/BPS samples and 15 normal bladder samples from 3 datasets (The GSE11783 dataset includes 5 non-ulcerated IC/BPS and 6 control samples, the GSE57560 contains 13 IC/BPS and 3 control tissues, the GSE621 contains 6 IC/BPS and 6 normal tissues). Dataset information is displayed in the Supplementary Tables 1–3.

### DEGs analysis of IC/BPS GEO datasets

We used robust multi-array averages (RMA) to calibrate and standardize the backgrounds of GSE11783, GSE57560 and GSE621 datasets. The batch effects between different datasets and irrelevant variables were eliminated by principal component analysis (PCA) using the “SVA Combat” package and visualized using the “ggplot” package in R software. The three GEO datasets were merged using Perl, and the data information remained unchanged. The “limma” package analyzed the DEGs, and the adjusted *P*-value <0.05 and absolute logFC (Fold change)>1 was used as the screening criteria for DEGs. Then, the “pheatmap” package presented the DEG expression in the datasets as a heatmap for hierarchical clustering analysis.

### Protein-protein interaction (PPI) and functional enrichment analysis of DEGs

We performed protein-protein interaction (PPI) and functional enrichment analysis on DEGs using the online analysis tool “String v11” (https://string-db.org/), (minimum required interaction score: 0.15).

### Immune-infiltration analysis

In order to understand the immune microenvironment of patients with IC/BPS, we analyzed the immune-infiltration of IC/BPS and normal tissues in the merged GEO dataset using the CIBERSORTX (https://cibersortx.stanford.edu) and visualization with R software.

### Clinical sample

From May 2021 to November 2021, we obtained 10 cases of IC/BPS bladder tissues and 10 cases of normal bladder tissues (from patients with bladder cancer). All patients with IC/BPS met the diagnostic criteria issued by NIDDK [4]. All patients corresponding to the samples signed the informed consent form. Accordance to the Helsinki Declaration, all experimental procedures related to patient samples have been reviewed and approved by the Ethics Committee of the Second Affiliated Hospital of Kunming Medical University (Approval NO. PJ-2021-70).

### Cell stimulation and transfection

Human mast cell HMC-1 was purchased from Otwo Biotech (Shenzhen, China). HCM-1 cells were cultured in DMEM medium (Gibco, USA) containing 10% fetal bovine serum, 100 U/mL penicillin and 0.1 g/mL streptomycin in an incubator at 37° C and 5% CO_2_ for routine use. For *in vitro* stimulation assay, HCM-1 cells were sensitized overnight with anti-TNP IgE antibody (final concentration of 2μg/mL). On the next day, after washing with PBS to remove excessive IgE antibody, cell pellet was resuspended in DMEM medium containing 2 mM L-glutamine, 10 μg/mL gentamicin, 10mM HEPES, 50 μM 2-ME, 50 ng/mL SCF and IL-3. The cell concentration was adjusted to 1×10^6^ cells/mL and seeded into a 24-well plate. The final concentration of 100 ng/mL OVA-TNP and 2 μM Ca2+ ionophore stimulated HCM-1 cells. After 24 hours of activation, the cell suspension of HCM-1 in each well was transferred to EP and centrifuged at 500 × g for 5 minutes at 4° C. And the supernatant and cells were used in subsequent experiments. Sh-BTK and negative control were designed and synthesized by RiboBio (Guangzhou, China). Transfection of sh-BTK or sh-NC was performed according to the instructions of the Lipofectamine 2000 kit (Thermo Fisher Scientific, USA). After 24 hours of transfection, the transfection efficiency was tested.

### Histopathological examination

Bladder tissue and normal bladder tissue of patients with IC/BPS were fixed with 4% paraformaldehyde and then embedded in paraffin. A paraffin microtome (HM355S; MICROM, Germany) was used to cut the embedded tissue into slices with a thickness of 4 μm. Then, according to the standard procedure of HE staining (Beyotime, Shanghai, China) and Masson staining kit (Solarbio, Beijing, China), tissue staining was performed. For IHC, after conventional dewaxing and rehydration of embedded tissue slices, the antigen was repaired with sodium citrate buffer for 15 minutes. After cooling to room temperature, the endogenous catalase of tissue slices were consumed with 3% H_2_O_2_, and then 5% BSA was added to block for 1 hour. Rabbit monoclonal mast cell tryptase antibody and BTK antibody (Abcam, UK) were added to the slices. After incubating overnight at 4° C, slices were added with Goat Anti-Rabbit IgG H&L (Abcam, UK) and incubated at room temperature for 1 hour. The DAB substrate kit (Abcam, UK) was used for color development. Histopathological changes of IC/BPS bladder tissue were observed under an optical microscope (BX53M, Olympus, Japan) after staining.

### Degranulation detection

For β-hexosaminidase quantification, the supernatant and cell pellet of stimulated mast cell were collected. Subsequently, 60 μL of supernatant and cell lysate were added to a 96-well ELISA plate and incubated with 60 μL of 4-nitrophenyl-N-acetyl-β-D-glucosamine (Sigma, USA) at 37° C for 2 hours. The reaction was quenched by adding 120 μL L-glycine (0.2 μM). Finally, the absorbance at 405 nm was detected with a microplate reader (Molecular Devices, USA). Histamine level in the supernatant of activated mast cells was analyzed by a Histamine kit (Labor Diagnostika Nord GmbH, Germany). For the measurement of tryptase activity, 20 μL supernatant was diluted with 80 μL of PBS, and 20 μL of substrate S-2288 (Chromogenix, China) was added. Then, the absorbance was measured at 405 nm every 30 seconds for 45 minutes with a microplate reader, and the Vmax value was expressed as mOD/min.

### Western blotting

RIPA protein lysate (Thermo Fisher Scientific, USA) was used to lyse bladder tissues and HMC-1 cells. After centrifugation at 12000 rpm for 15 minutes at 4° C, the concentration of protein was measured by BCA kit (Beyotime, Shanghai, China). Protein was added to the polyacrylamide gel for electrophoresis, and then protein on the gel was transferred to the polyvinylidene fluoride membrane (PVDF). After blocking with 5% BSA for 1 hour, rabbit monoclonal BTK (1:1000; Abcam, UK) was added to the membrane. After incubating overnight at 4° C, Goat Anti-Rabbit IgG H&L was added to the membrane and incubated for 1 hour at room temperature. The ECL substrate kit was used for the colour development of the band. Gel imager (Gel Doc XR+; Bio-Rad, USA) was used for observation and photography. Image J software (Version 1.0; National Institutes of Health, US) analyzed the grey value of the band and calculated the relative expression level of the protein.

### Wound-healing assay

In short, after trypsinization of HMC-1 cells in each group in the logarithmic growth phase, the cell concentration was adjusted to 5×10^5^ and inoculated into 96-well plates. After incubating at constant temperature for 24 hours, the lower and central part of the 96-well plate was aligned with a scratch tester and gently pushed upward to form scratches. Then, the plate was put into an incubator at 37° C and 5% CO2 for constant temperature cultivation. The culture system was 100 μL/well. Cells were seeded at 0 hour and 24 hours with a Celigo cell imaging analyzer (Nexcelom, USA) for observation and photographing, and the migration area was analyzed and the Wound-Healing rate was calculated by Image J software.

### Transwell assay

After dilution of Matrigel (medium: Matrigel=3:1; BD, USA), 200 μL Matrigel was coated in the upper chamber of Transwell (Corning Incorporated, USA) and dried at 4° C. HMC-1 cells in the logarithmic growth phase were treated with conventional trypsin, then resuspended in serum-free medium, and the cell density was adjusted to 1×10^6^ cells/mL. After being cultured in an incubator for 24 hours, it was fixed with 4% paraformaldehyde for 15 minutes. After staining and washing, the lower chamber was observed and photographed under an optical microscope, and the invasion cells were counted by Image J software.

### Statistical analysis

GraphPad Prism (Version 8.0) is used for data analysis and graphing. For pairwise comparisons, a t-test was used, and one-way analysis of variance was performed for multiple group comparisons. *P*<0.05 was considered statistically significant.

### Data availability

The GSE11783, GSE57560, and GSE621 datasets generated and/or analysed during the current study are available in the GEO database repository, (https://www.ncbi.nlm.nih.gov/guide/genes-expression/). And original photographs of Western blots in Figures 4E, 5A, and sh-BTK and NC sequences are shown in Supplementary Figures 1, 2.

## Supplementary Material

Supplementary Figures

Supplementary Tables
