# Supplementary material for: Bruton tyrosine kinase (BTK) may be a potential therapeutic target for interstitial cystitis/bladder pain syndrome
Source: Aging (Albany NY). 2022 Sep 5;14(17):7052–64. doi: 10.18632/aging.204271 (PMC9512503; doi:10.18632/aging.204271)
Supplement: Supplementary Figures [file aging-14-204271-s001.pdf]

SUPPLEMENTARY FIGURES

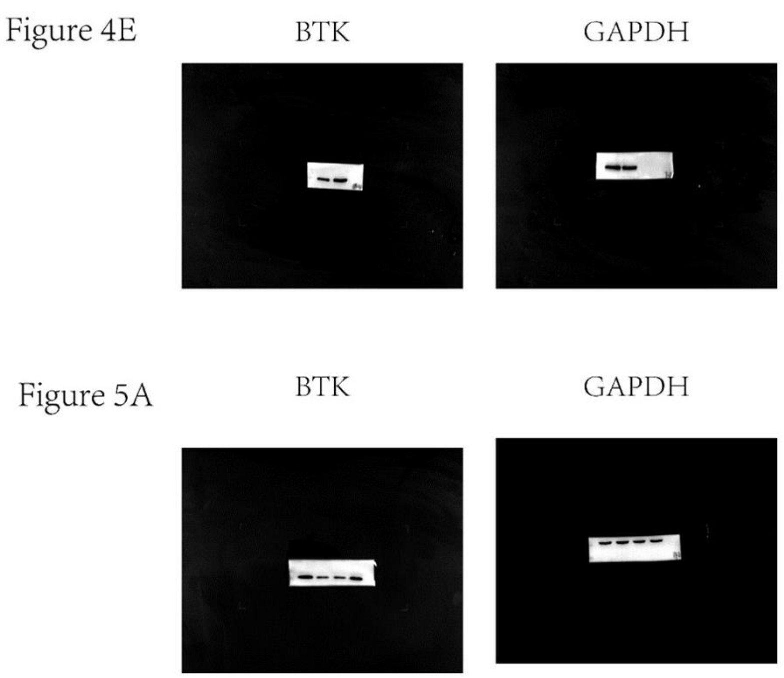

Supplementary Figure 1. Original photo of Western bloting of Figures 4E, 5A.

sh-BTK#1

5'- CACCGCTGGACTCTTGAGTGTAAAGCGAACTTTACACTCAAGAGTCCAGC -3'

3'- CGACCTGAGAACTCACATTTCGCTTGAAATGTGAGTTCTCAGGTCGAAAA -5'

sh-BTK#2

5'- CACCGCACTGAACCTTTCCTCAACACGAATGTTGAGGAAAGGTTCAGTGC -3'

3'- CGTGACTTGGAAAGGAGTTGTGCTTACAACTCCTTTCCAAGTCACGAAAA -5'

sh-BTK#3

5'- CACCGCTCACAACCATCTGTAATGGCGAACCATTACAGATGGTTGTGAGC -3'

3'- CGAGTGTTGGTAGACATTACCGCTTGGTAATGTCTACCAACACTCGAAAA -5'

NC 5′-GATCCACTACCGTTGTTATAGGTGGAGTACTGCACCTATAACAACGGTAGTTTTTTC-3′

Supplementary Figure 2. The sh-BTK and NC sequences used in our cell verification experiment.
