# Supplementary material for: Bruton tyrosine kinase (BTK) may be a potential therapeutic target for interstitial cystitis/bladder pain syndrome
Source: Aging (Albany NY). 2022 Sep 5;14(17):7052–64. doi: 10.18632/aging.204271 (PMC9512503; doi:10.18632/aging.204271)
Supplement: Supplementary Tables [file aging-14-204271-s002.pdf]

SUPPLEMENTARY TABLES

Supplementary Table 1. Information of the three GEO datasets.

	GSE11783	GSE57560	GSE621
**References**	Gamper et al. [1]	Colaco et al. [2]	Keay et al. [3]
**Platform**	Affymetrix Human Genome U133 Plus 2.0 Array	Agilent-039494 SurePrint G3 Human GE v2 8x60K Microarray 039381	Res Gen Human
**IC/BPS**	5 samples (GSM298205, GSM298207, GSM298210, GSM298213, GSM402541)	13 samples (GSM1384758, GSM1384759, GSM1384760, GSM1384761, GSM1384762, GSM1384763, GSM1384764, GSM1384765, GSM1384766, GSM1384767, GSM1384768, GSM1384769, GSM1384770)	6 samples (GSM4885, GSM4887, GSM4889, GSM4894, GSM4895, GSM4896)
**Normal**	6 samples (GSM298203, GSM298204, GSM298212, GSM298215, GSM298216, GSM298217)	3 samples (GSM1384771, GSM1384772, GSM1384773)	6 samples (GSM4886, GSM4888, GSM4890, GSM4891, GSM4892, GSM4893)

Supplementary Table 2. Basic characteristics of patients.

	GSE11783		GSE57560		GSE621	
Group	IC/BPS patients	Control	IC/BPS patients	Control	IC/BPS patients	Control
No.	5	6	13	3	6	6
Gender	female	female	female	female	-	-
Race	white	white	-	-	-	-
Age [year], mean, SD	78.0, 7.5	59.2, 13.2	43.8, 16.7	57.3, 16.9	>18	>18

Supplementary Table 3. DEGs with adj. *P*-values < 0.05 and |$\log_2$FC| > 2.

ID	$\log_2$FC	*P*. Value	adj. *P*-values
AQP9	2.77	2.79E-07	9.06E-04
CFP	1.93	6.51E-06	1.06E-02
S100A8	2.34	2.57E-05	2.78E-02
CD37	1.94	4.08E-05	3.31E-02
BTK	1.73	6.30E-05	4.09E-02

REFERENCES

1. Gamper M, Viereck V, Geissbühler V, Eberhard J, Binder J, Moll C, Rehrauer H, Moser R. Gene expression profile of bladder tissue of patients with ulcerative interstitial cystitis. BMC Genomics. 2009; 10:199. https://doi.org/10.1186/1471-2164-10-199 PMID:19400928

2. Walker SJ, Colaco M, Koslov DS, Keys T, Evans RJ, Badlani GH, Andersson KE. Transcriptome analysis of bladder biopsy from interstitial cystitis/bladder pain syndrome patients. Genom Data. 2014; 2:366–8. https://doi.org/10.1016/j.gdata.2014.10.009 PMID:26484132

3. Keay S, Seillier-Moiseiwitsch F, Zhang CO, Chai TC, Zhang J. Changes in human bladder epithelial cell gene expression associated with interstitial cystitis or antiproliferative factor treatment. Physiol Genomics. 2003; 14:107–15. https://doi.org/10.1152/physiolgenomics.00055.2003 PMID:12847144
